# Parental Migration and Psychological Well-Being of Children in Rural China

**DOI:** 10.3390/ijerph18158085

**Published:** 2021-07-30

**Authors:** Rui Chen, Li Zhou

**Affiliations:** College of Economics and Management, Nanjing Agricultural University, Nanjing 210095, China; ccccruii@163.com

**Keywords:** parental migration, depression, subjective happiness, mediating effect, absolute and relative poverty

## Abstract

This paper empirically analyzes the impact of parental migration on the psychological well-being of children using ordered probit models based on a survey conducted among 1680 primary school students and their parents in Majiang County, Guizhou Province, China in 2020. The findings are as follows. First, compared with having no migrant parents, having two migrant parents significantly reduces the psychological well-being of children and having one migrant parent has no significant effect. Second, mediation analysis shows that parental migration reduces child depression by increasing household absolute and relative incomes. It also increases depression and reduces the subjective happiness of children by reducing parental discipline. However, it has no significant impact on parent–child interactions. Third, by dividing the sample by absolute and relative poverty, we find that the effect of parental migration on the psychological well-being of children varies with household economic conditions. Comparatively speaking, children from poor households are more affected by parental migration in terms of depression, whereas children from non-poor households are more affected by parental migration in terms of subjective happiness. This paper examines the transmission mechanism between parental migration and the psychological well-being of children, provides a perspective of household economic conditions for child psychology and offers useful insights for family education and government policymaking in this area.

## 1. Introduction

China’s rapid economic growth and urbanization have promoted massive migration of rural residents to cities since the early 1980s. According to the National Bureau of Statistics of China, there were 290 million migrant workers nationwide at the end of 2020. A majority (73.4%) of them were aged 21–50 years. However, it is difficult for migrant workers to have a stable life due to the household registration system. Moreover, the cost of raising their children in cities is too high. Therefore, most migrant workers have to leave their children in their villages in the care of others, resulting in parental absence. According to the Ministry of Civil Affairs of the People’s Republic of China, there were approximately 6.436 million rural left-behind children in China in 2020 (Children under the age of 18 years with one or two migrant parents).

Parental absence not only prevents rural children from experiencing the social and economic benefits available to urban children but may also affect their psychological health and well-being. On the one hand, migrant parents are less able to conduct high-quality parent–child interactions due to life pressures or working time constraints, reflecting the negative effect of parental migration through parental companionship. According to the attachment theory of Bowlby [1], long-term separation of children from the attached person is likely to cause emotional distress in children, such as anxiety, anger, and depression. Li and Zang [2] and Rebicova et al. [3] suggest that parental absence makes children more likely to be exposed to danger, causing emotional problems, and even externalized behavioral risks.

On the other hand, parental migration alleviates household economic constraints to a certain extent, reflecting the positive effect of parental migration through household income. Improvements in household income have been shown to reduce a children’s risk of facing discrimination or bullying [4,5,6], increase the possibility of children participating in extracurricular activities and eliminating negative emotions [7,8,9], or have a positive psychological effect on children through the neighborhood effects [10,11,12], thereby improving their psychological well-being.

However, no consensus has emerged on the impact of parental migration on the psychological well-being of children. Some researchers believe that parental migration significantly reduces children’s psychological well-being. Compared with non-left-behind children, left-behind children are more likely to experience negative emotions, such as loneliness, guilt, and despair [13,14], and have lower psychological resilience [15]. In terms of social psychology, left-behind children have lower self-esteem [16] and poorer interpersonal relationships [17]. Other researchers argue that the psychological role of parental migration is exaggerated. For example, Ren and Treiman [18] conclude that there is no difference in psychosocial health between left-behind and non-left-behind children based on data from China Family Panel Studies (CFPS). Nguyen [19] comparatively analyzes parental migration in Ethiopia, India, Peru, and Vietnam, and finds that the impact of parental migration on children varies among countries and that parental migration does not have a significant impact on children in Ethiopia.

Most previous studies examine the difference between left-behind and non-left-behind children from the psychological and sociological perspectives but do not investigate the causal relationship between parental migration and the psychological status of children [20]. Some studies discuss this relationship using an empirical model but do not clarify the mechanism behind it [21]. In addition, due to the easier identification of behavioral disorders, most studies only assess the psychological status of rural children from an objective perspective by measuring the presence of self-harm [22] but do not evaluate their subjective feelings. Therefore, we empirically analyze the impact of parental migration on the psychological well-being of rural children and the mechanism underlying it using the ordered probit model with depression and subjective happiness as measured. Data for these variables were taken from a survey conducted by Nanjing Agricultural University among 1680 students in Majiang County, Guizhou Province, China, in 2020.

The main contributions of this paper are as follows. First, we assess the psychological well-being of children from both negative and positive perspectives based on comprehensive subjective self-evaluation data, such as depression and subjective happiness. Second, we examine the causal relationship between different types of parental migration, including “neither parent migrated”, “one parent migrated”, and “both parents migrated”, and the psychological well-being of children. Third, we investigate the impact of parental migration through parental companionship and household income from the perspectives of absolute and relative income, parent–child interactions, and parental discipline, respectively. Fourth, we examine the impact of parental migration on children under different household economic conditions by distinguishing between absolute and relative poverty.

The paper proceeds as follows. Section 2 presents the data, variables, and model design. Section 3 reports the empirical results, including benchmark model results, mechanism analysis, and heterogeneity analysis. Finally, Section 4 offers conclusions.

## 2. Data, Variables, and Model Specification

### 2.1. Participants and Procedures

The data used in this study come from the baseline survey of the “Sixth Power” Educational Poverty Alleviation Program of the TREE research team of Nanjing Agricultural University, conducted in September 2020. The respondents were fifth-grade children and their parents from 21 primary schools in Majiang County, Guizhou Province. A total of 1800 questionnaires were administered to children and 1800 to their parents. Gosling et al. [23] point out that in the field of psychology, self-administered online questionnaires provide the same statistical results as paper questionnaires and will not be adversely affected by inadvertent or repeated answers. Therefore, the research team designed two separate online questionnaires: one for children and the other for their parents. The research team got in touch with the class teachers through the Department of Education and Science of Majiang County. With their assistance, the children and their parents were informed in detail of the purpose and process of the survey. After signing the informed consent form, they were instructed to complete the questionnaire on their own. Finally, 1680 questionnaires were collected from children and 1680 from their parents, a response rate of 93.33%.

### 2.2. Measurements

#### 2.2.1. Demographics of Children and Their Parents

The questionnaire for children included questions about their gender, whether they are an only child, whether they reside at the school, whether they have transferred to another school, whether they are class leaders, and whether they were sick in the last month. The parent questionnaire collected information about parental marital status, parental education (1 = no school, 2 = primary school, 3 = junior high school, 4 = vocational high school or technical secondary school, 5 = high school, 6 = junior college, 7 = university and above), and household size.

#### 2.2.2. Parental Migration

Existing studies usually define migration as working outside the place of residence for 6 months or more. However, this definition may underestimate the impact of parental migration on the quality of parental companionship. Therefore, we measure parental migration by children’s self-reports. To this end, children were asked the question “Do either of your parents work outside? (It means they work outside of Majiang County and cannot return home every day)”, with the options of “1 = both parents; 2 = father only; 3 = mother only; 4 = neither”. Accordingly, we divide parental migration into three categories: “both parents migrated”, “one parent migrated”, and “neither parent migrated”.

#### 2.2.3. Psychological Well-Being

We use depression as a negative measure of psychological well-being. To this end, eight questions from the Children’s Depression Inventory (CDI, “Er Tong Yi Yu Liang Biao” in Chinese version) (CDI was developed and revised by Kovacs [24]. It is the first questionnaire to measure child depression and has high reliability and validity. It applies to children aged 7–17 years, requires low reading proficiency, and takes under 15 min to complete. Yu and Li [25] were the first to introduce the inventory to China; they translated it and verified its feasibility by taking primary and junior high school students as research objects. So far, Chinese Scholars have conducted a large number of tests using this approach on domestic children, and the inventory has been fully validated in the Chinese context) were included in the questionnaire. To determine the occurrence frequency of depression, three answer options were provided for each question (e.g., “occasionally unhappy”, “often unhappy”, and “always unhappy”). A score of 0 was given for “occasionally”, 1 was given for “often”, and 2 was given for “always”. The higher the total score, the more severe the depression.

We also use subjective happiness as a positive measure of psychological well-being. It was assessed using the question, “Do you think you are happy?”, with ratings scoring from one (very unhappy) to ten (very happy).

**Figure 1:**
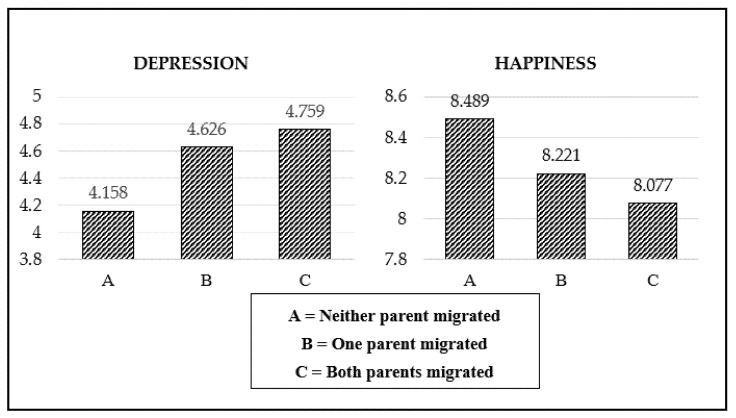
Comparison of children’s psychological well-being for different types of parental migration.

Figure 1 shows the psychological well-being of children with different parental migration statuses. The vertical axis represents the average score of depression or subjective happiness, and the horizontal axis represents the parental migration status. As shown in Figure 1, children with one and especially two migrant parents had more severe depression and lower subjective happiness.

#### 2.2.4. Parental Companionship

As mentioned above, parental migration may reduce children’s psychological well-being by reducing parental companionship. In this paper, we measure the quality of parental companionship by parent–child interactions and parental discipline. To assess parent–child interactions, children were asked six questions: “How often does your mother/father discuss with you what happened at school/your relationship with friends/your concerns or worries?”, with three response options: “never”, “sometimes”, and “always”. The responses were assigned a score of 1, 2, and 3, respectively, to calculate the total scores of mother–child and father–child interactions to represent parent–child interactions. To assess parental discipline, children were asked two questions: “How strict are your parents with you regarding the time spent surfing the Internet or playing with mobile phones/the time spent watching TV?”, with three response options: “no discipline”, “mild discipline”, and “severe discipline”. The responses were assigned a score of 1, 2, and 3, respectively, to calculate the total score.

#### 2.2.5. Household Income

Parental migration may also improve the psychological well-being of children by increasing household income. In this regard, parents reported gross household income in the previous year by answering, “How much was your total household income in the last year (without deducting expenses)?” We divide the household income into absolute and relative income measures. Absolute income is measured by the household’s per capita annual income. After excluding outliers that were less than or equal to 0, the reported gross household income is divided by household size and expressed in logarithm form. Relative income is defined as the logarithmic difference between the actual per capita income of the surveyed households and the per capita disposable income of the county in 2020, i.e., $lny_{i}-ln\bar{y_{g}}$, where $\bar{y_{g}}$ is the per capita disposable income of urban or rural residents in Majiang County (According to the Statistical Bulletin of National Economic and Social Development of Majiang County in 2020, the per capita disposable income of urban and rural residents in Majiang County in 2020 was 33,290 and 10,714 yuan respectively, which was about 4424.72 and 1552.78 USD calculated according to the average exchange rate of the year. URL: http://www.majiang.gov.cn/zfxxgk/fdzdgknr/ghjh_5765101/gmjjhshfzgh/202104/t20210414_67798664.html, accessed on 20 June 2021). Urban and rural residents are distinguished by the registered residence of the household head.

#### 2.2.6. Absolute and Relative Poverty

Household economic conditions have been a long-standing topic of concern in psychology. Therefore, following previous research, we divide the sample by absolute and relative poverty from the perspective of household economic conditions in the analysis of heterogeneity. (a) Absolute poverty: In 2013, targeted poverty alleviation was carried out nationwide in China to register poor households in each village by county and develop assistance measures on a household-by-household and village-by-village basis. Households with a per capita net income lower than or equal to 2736 yuan, which was the national rural poverty line in 2013, were registered as poor households for assistance. In the parent questionnaire, we use the question, “Is your household a registered low-income household (poor household)?”, to identify absolute poverty. (b) Relative poverty: Following the theory of Fuchs [26], we use 50% of the median household income per capita in the sample as the relative poverty line. In other words, relative poverty is defined as per capita household income less than or equal to the relative poverty line.

### 2.3. Statistical Analysis

Considering that most primary schools in Majiang County are boarding schools, and that teaching and management practice may vary across schools, the psychological well-being of children is likely to be affected by unobservable variables in school life. Therefore, we control the school fixed effects in the following regression models to eliminate the inherent differences in school life. Outliers were treated as missing values.

**Figure 2:**
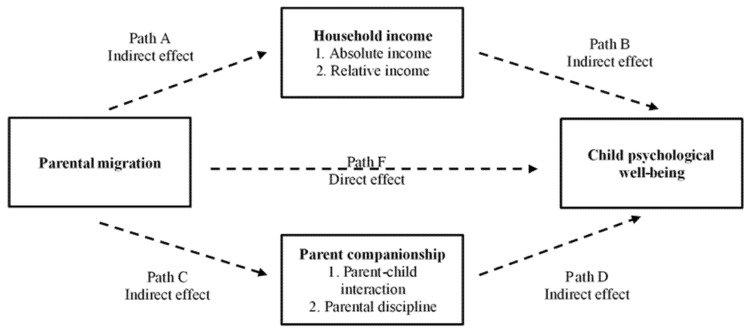
Analytic framework of mediating effect.

**Figure 3:**
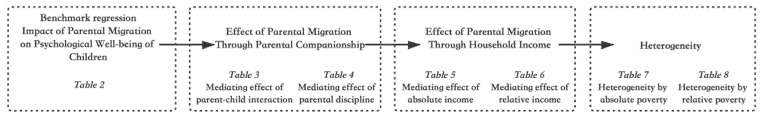
Overall analytical procedure.

First, we use an ordered probit model to identify the causal relationship between parental migration and the psychological well-being of children. Second, we examine the two ways (parental companionship and household income) that parental migration affects the psychological well-being of children using four variables, namely parent–child interactions, parental discipline, absolute and relative incomes in the previous year, based on the mediation model [27]. The analysis framework is shown in Figure 2. Finally, we divide the sample by absolute and relative poverty to analyze the heterogeneity based on the ordered probit model from the perspective of household economic conditions. Figure 3 provides an overview of the analysis procedure.

## 3. Results

### 3.1. Descriptive Statistics

The descriptive statistics are provided in Table 1. In this study, children with one, two, or no migrant parents accounted for 28.51%, 26.63%, and 42.86% of respondents, respectively. Among them, 895 (53.27%) were boys and 785 (46.73%) were girls.

### 3.2. Impact of Parental Migration on Psychological Well-Being of Children

The benchmark regression results are presented in Table 2. School fixed effects are controlled in Models (2), (4), (6), and (8), but not in Models (1), (3), (5), and (7). The regression results show that compared with having no migrant parents, having two migrant parents has a significant positive impact on child depression and a significant negative impact on their subjective happiness. However, having one migrant parent, i.e., having a migrant mother only or having a migrant father only, has no significant impact on the psychological well-being of children. After controlling for the school fixed effects, both the positive and negative impacts of parental migration on the psychological well-being of children increases, resulting in an increased significance, which confirms the robustness of the estimates.

### 3.3. Effect of Parental Migration through Parental Companionship

The results of the mediating effect of parent–child interactions are presented in Table 3 In both Models (3) and (6), the coefficient of parent–child interactions is significant at the 1% level, indicating that it significantly reduces children’s depression and improves their subjective happiness. In Models (2) and (5), however, parent–child interactions are not significantly affected by parental migration, suggesting that this variable does not have a mediating effect. Considering the definition of parent–child interactions in this study, this may reflect the widespread availability of Internet access and affordable mobile devices that enable migrant parents to keep in touch with their children. Hence, parental migration would not affect the psychological well-being of children by reducing parent–child interactions.

The regression results of parental discipline are given in Table 4. The coefficients of key variables of the three regression equations all passed the significance test at 5% or 1%. This indicates that parental migration significantly reduces the quality of parental discipline, thereby increasing children’s depression and reducing their subjective happiness. In summary, parental migration influences the depression and subjective happiness of children through parental companionship, which is mainly observed for parental discipline.

### 3.4. Effect of Parental Migration through Household Income

The results of the mediating effect of absolute income are presented in Table 5. The results of Models (1), (2), and (3) show that parental migration significantly (at the 1% level) increases absolute per capita household income, thereby significantly reducing child depression. However, the coefficient of absolute per capita income in Model (6) did not pass the significance test. That finding indicates that absolute income does not play a mediating role in the impact of parental migration on children’s subjective happiness. The results of the mediating effect of relative income are presented in Table 6. The coefficients of relevant variables in Models (7), (8), and (9) passed the significance test at 5% or 1%. This indicates that relative income plays a mediating role in the impact of parental migration on children’s depression. However, the results of Model (12) suggest that parental migration does not affect the psychological well-being of children through the increase of relative household income in terms of subjective happiness.

Overall, although children are usually not participants in the household economy, parental migration influences child depression through household income. We speculate that child depression is a psychological variable related to material conditions. In this case, parental migration can reduce child depression in two ways: (a) by improving household economic conditions, that is, absolute income, to improve children’s objective living conditions; and (b) by improving household economic status, that is, relative income, which may make children feel more respected by their peers, thereby improving their subjective social status. However, the absolute and relative income is not mediating variables between parental migration and children’s subjective happiness. This indicates that child happiness may be more dependent on other spiritual parent–child factors. During the survey, some children said that they were happiest when their parents returned home during the Spring Festival.

### 3.5. Heterogeneity

Heterogeneity by absolute poverty is discussed in Table 7. We find that having two migrant parents has a significant positive effect at the 5% level on child depression among households in absolute poverty, but not among those not in absolute poverty. In terms of subjective happiness, it has no significant effect among households in absolute poverty, and a significant negative effect at the 5% level among those not in absolute poverty.

Heterogeneity by relative poverty is depicted in Table 8. Ordered probit regression reveals that having one or two migrant parents significantly increases child depression at the 5% and 1% levels, respectively, among households in relative poverty, but does not affect their subjective happiness. Among households not in relative poverty, only having two migrant parents has a significant negative effect on children’s psychological well-being.

The heterogeneity analysis shows that the impact of parental migration on child depression varies with household economic conditions. Comparatively speaking, children from poor households, whether in absolute or relative poverty, are more affected by parental migration in terms of depression, whereas children from non-poor households are more affected by parental migration in terms of subjective happiness.

## 4. Discussion

In rural areas of China, it is common for young and middle-aged adults to migrate to cities for employment and leave their children in their hometowns in the care of others. Although the relationship between parental migration and children’s psychological well-being has been extensively studied, no consensus has yet emerged. This lack of consensus may reflect the fact that children’s psychological well-being is a complex phenomenon involving many measures, and different psychological variables may be affected differently by parental migration. Motivated by this conjecture, we measure the psychological well-being of children from the negative and positive aspects using depression and subjective happiness, respectively, and test the mediating effect of parental migration on the psychological well-being of children based on the mediation model.

First, we examine the impact of parental migration on children’s depression and subjective happiness based on a survey among 1680 primary school students and their parents in Majiang County, Guizhou Province. We find that compared with having no migrant parents, having two migrant parents significantly reduces the psychological well-being of children, but having one migrant parent has no significant effect.

Second, we investigate the impact of parental migration on the psychological well-being of children through parental companionship and household income from the perspectives of absolute and relative income, parent–child interactions, and parental discipline, respectively. This is the most unique and important contribution of this study. We find that having two migrant parents increases child depression mainly by reducing the quality of parental discipline, and it significantly reduces child depression by increasing household income, including absolute and relative income. However, parental migration does not influence children’s subjective happiness through household income or parent–child interactions but does exert an influence mainly through parental discipline. Based on the previous analysis, we believe that the negative effect of parental migration through parental companionship is greater than the positive effect through household income. In other words, although parental migration improves the household material conditions and economic status, it is detrimental to the healthy psychological development of children by affecting parental companionship.

This finding confirms our conjecture made above that some psychological variables of children are affected differently by parental migration. We also emphasize the role of parental discipline in the growth of children. Similarly, Dickson et al. [28] find a strong correlation between parental discipline and child alcoholism among Swedish girls. A systematic review by Flanagan et al. [29] also demonstrates the important role of parental discipline in juvenile delinquency. These studies provide credible support for our conclusion. In addition, subjective happiness is often considered to be affected by relative income in happiness economics [30,31,32]. However, our empirical results demonstrate that relative income is not a mediating variable between parental migration and children’s subjective happiness. This finding provides a useful addition to the existing literature. Finally, we believe that parental migration does not significantly affect the quality of parent–child interactions. This argument is supported by the existence of well-developed communication networks in China.

This study also provides a perspective on the impact of household economic conditions in terms of child psychology. When dividing the sample by absolute and relative poverty, ordered probit regression reveals that the effect of parental migration on the psychological well-being of children varies with household economic conditions. Comparatively speaking, children from poor households are more affected by parental migration in terms of depression; whereas children from non-poor households are more affected by parental migration in terms of subjective happiness. This phenomenon is reasonable because people of different social classes may have different concerns and different patterns of emotional responses [33].

This study supplements the existing literature on the impact of parental migration on the psychological well-being of rural children. However, some limitations need to be considered when interpreting the results. First, the data used in this study are cross-sectional data, but parental migration may be affected by unobservable household factors, causing endogeneity problems. Therefore, instrumental variables or a difference-in-difference approach with panel data should be used in future research to better examine the causal relationship between parental migration and children’s psychological well-being. Second, due to space limitations in the questionnaire, the depression variables used in the study are obtained by quantifying the eight questions in the CDI. However, the CDI also uses value assignment and defines the level of depression by the total score. Therefore, the variables used in this study still hold scientific value. Finally, the sample in this study comprised only fifth-grade students in Majiang County, Guizhou Province, China. Although the sample size is large, there may be a selectivity bias, which would limit the representativeness of the sample. In future research, it is expected to conduct a survey with a larger scope and strict random sampling.

## 5. Conclusions

To conclude, this study reveals the influence of different types of parental migration on children’s psychological well-being and the transmission mechanism through parental companionship and household income. The results also incorporate the perspective of household economic conditions into the study of child psychology.

Despite some limitations, this study provides useful insights for family education and government policymaking. For parents in rural China, migration is a result of deliberation and is hard to change. However, continuity and persistence in terms of parental discipline will be helpful. This study identifies that migration will reduce the psychological well-being of children through low-quality parental discipline. Thus, it will be effective for parents to improve children’s mental health by strengthening the management of their children’s education and daily life. From the perspective of public policies, the government can also take measures to improve rural children’s psychological well-being. In areas where laborers have migrated from, the government can set up children centers in the village and regularly organize psychological counseling or other such activities. Government should also aim to develop the local economy. This will be the most fundamental way to solve the parent–child separation problem, as a developed local economy allows parents to work locally and earn sufficient income. As for the areas with laborer inflows, the government could make it easier for rural children to live with their migrated parents by ensuring their medical care and reducing the cost of school enrollment.

In addition, changes to the household registration system could be made. In the areas with laborer inflows, having a non-local household registration restricts migrant workers from buying a house or obtaining the same social security as local residents. In turn, this increases their risk of unemployment and can even lead to discrimination. Although currently, the government is gradually reforming and weakening the household registration system, further progress could be made. Policies introduced in some cities tend to attract only high-tech talent, which is not conducive to achieving balance. Therefore, the government could not only ensure social security, including medical care, employment security, the cost of school enrollment, and the quality of education of migrant workers and their children but also loosen the restrictions for settlement. Such looser restrictions would encourage the free flow of people with different skills and from a range of occupational backgrounds, which will be conducive to attaining a balance between the well-being of the economy and the population, and to solving the parent–child separation problem as well.

## Figures and Tables

**Table 1:** Demographic characteristics of the sample.

Variables	Overall	One Parent Migrated	Both Parents Migrated	Neither Parent Migrated
*N*	1680 (100%)	479 (28.51%)	481 (28.63%)	720 (42.86%)
Dependent variables				
Depression (M±SD)	4.46 ± 2.52	4.63 ± 2.50	4.76 ± 2.68	4.16 ± 2.38
Subjective happiness (M±SD)	8.29 ± 2.14	8.22 ± 2.23	8.08 ± 2.21	8.49 ± 2.01
Independent variables				
Absolute income (M±SD)	−0.85 ± 1.00	−0.92 ± 0.95	−0.83 ± 0.94	−0.81 ± 1.08
Relative income (M±SD)	−1.01 ± 0.95	−1.03 ± 0.94	−0.93 ± 0.93	−1.04 ± 0.98
Parent-child interactions (M±SD)	12.21 ± 2.84	12.08 ± 2.81	12.10 ± 3.01	12.36 ± 2.73
Parental discipline (M±SD)	5.02 ± 1.07	5.08 ± 1.02	4.78 ± 1.20	5.15 ± 0.90
Controls				
Gender				
Child is boy	895 (53.27%)	259 (54.07%)	248 (51.56%)	388 (53.89%)
Child is girl	785 (46.73%)	220 (45.93%)	233 (48.44%)	332 (46.11%)
Only child in family	233 (13.87%)	66 (13.78%)	72 (14.97%)	95 (13.19%)
Boarder	637 (44.08%)	177 (42.55%)	241 (59.65%)	219 (35.04%)
Transfer student	311 (21.52%)	100 (24.04%)	85 (21.04%)	126 (20.16%)
Class leader	419 (24.94%)	102 (21.29%)	86 (17.88%)	231 (32.08%)
Fell ill last four weeks	118 (8.17%)	35 (8.41%)	30 (7.43%)	53 (8.48%)
Parents divorced/separated	275 (19.03%)	103 (24.76%)	101 (25.00%)	71 (11.36%)
Education mother				
High school and above (including vocational and secondary)	218 (15.09%)	51 (12.26%)	34 (8.42%)	133 (21.28%)
Education father				
High school and above (including vocational and secondary)	226 (15.64%)	46 (11.06%)	39 (9.65%)	141 (22.56%)
Household size (M±SD)	5.15 ± 1.54	4.99 ± 1.45	5.56 ± 1.76	4.99 ± 1.39

**Table 2:** Impact of parental migration on children’s psychological well-being.

	(1)	(2)	(3)	(4)	(5)	(6)	(7)	(8)
	Depression				Subjective Happiness			
One parent migrated	0.073	0.085	--	--	−0.069	−0.078	--	--
	(0.065)	(0.066)	--	--	(0.072)	(0.074)	--	--
Both parents migrated	0.137 **	0.150 **	0.138 **	0.151 **	−0.129 *	−0.180 **	−0.130 *	−0.181 **
	(0.069)	(0.070)	(0.069)	(0.070)	(0.075)	(0.077)	(0.075)	(0.077)
Only mother migrated	--	--	0.169	0.176	--	--	−0.130	−0.151
	--	--	(0.110)	(0.111)	--	--	(0.120)	(0.122)
Only father migrated	--	--	0.043	0.057	--	--	−0.049	−0.055
	--	--	(0.071)	(0.072)	--	--	(0.079)	(0.080)
Gender	0.001	0.008	0.000	0.007	−0.152 **	−0.164 ***	−0.152 **	−0.164 ***
	(0.054)	(0.055)	(0.054)	(0.055)	(0.060)	(0.061)	(0.060)	(0.061)
Only child in family	0.109	0.118	0.111	0.119	0.042	0.038	0.041	0.036
	(0.082)	(0.083)	(0.082)	(0.083)	(0.090)	(0.091)	(0.090)	(0.091)
Boarder	0.099 *	0.103	0.097 *	0.099	−0.045	0.003	−0.044	0.006
	(0.056)	(0.067)	(0.057)	(0.067)	(0.062)	(0.074)	(0.062)	(0.074)
Transfer student	−0.061	−0.008	−0.055	−0.004	−0.019	−0.062	−0.023	−0.065
	(0.065)	(0.073)	(0.065)	(0.073)	(0.071)	(0.080)	(0.071)	(0.080)
Class leader	−0.290 ***	−0.307 ***	−0.289 ***	−0.306 ***	0.170 **	0.195 ***	0.170 **	0.195 ***
	(0.062)	(0.064)	(0.062)	(0.064)	(0.069)	(0.071)	(0.070)	(0.071)
Fell ill last four weeks	0.087	0.080	0.087	0.079	−0.038	−0.011	−0.037	−0.010
	(0.097)	(0.098)	(0.097)	(0.098)	(0.106)	(0.108)	(0.106)	(0.108)
Parents divorced/separated	0.100	0.067	0.097	0.063	−0.280 ***	−0.242 ***	−0.277 ***	−0.238 ***
	(0.073)	(0.073)	(0.073)	(0.073)	(0.078)	(0.079)	(0.078)	(0.079)
Education mother	0.033	0.041	0.035	0.043	−0.138	−0.143	−0.139	−0.145
	(0.086)	(0.089)	(0.086)	(0.089)	(0.093)	(0.096)	(0.093)	(0.096)
Education father	−0.094	−0.096	−0.094	−0.096	−0.037	−0.046	−0.037	−0.046
	(0.085)	(0.087)	(0.085)	(0.087)	(0.093)	(0.095)	(0.093)	(0.095)
Household size	−0.004	−0.008	−0.004	−0.007	−0.009	−0.007	−0.009	−0.007
	(0.018)	(0.018)	(0.018)	(0.018)	(0.020)	(0.020)	(0.020)	(0.020)
School fixed effects	no	yes	no	yes	no	yes	no	yes
*N*	1445	1445	1445	1445	1445	1445	1445	1445
chi2	46.925	88.109	48.092	89.133	38.382	91.285	38.794	91.847

Note: * *p* < 0.1, ** *p* < 0.05, *** *p* < 0.01. Numbers [(1)–(8)] at the top row signify the serial numbers of the empirical analysis model. In the regression, we used Depression and Subjective Happiness as dependent variables. The higher the depression score, the more serious problem present in a child’s psychology, whereas the subjective happiness score is the opposite. Variables in the leftmost column are independent variables (One parent migrated, Both parents migrated, Only the mother migrated, and Only the father migrated) or controls (from Gender to Household size). The Tables in the rest of this paper use the same conventions.

**Table 3:** Mediating effect of parent–child interactions.

	(1)	(2)	(3)	(4)	(5)	(6)
	Depression	Parent–Child Interactions	Depression	Subjective Happiness	Parent–Child Interactions	Subjective Happiness
One parent migrated	0.085	−0.021	0.084	−0.078	−0.021	−0.079
	(0.066)	(0.067)	(0.066)	(0.074)	(0.067)	(0.074)
Both parents migrated	0.150 **	−0.030	0.147 **	−0.180 **	−0.030	−0.178 **
	(0.070)	(0.070)	(0.070)	(0.077)	(0.070)	(0.077)
Parent-child interactions	--	--	−0.102 ***	--	--	0.074 ***
	--	--	(0.010)	--	--	(0.011)
Controls	yes	yes	yes	yes	yes	yes
School fixed effects	yes	yes	yes	yes	yes	yes
*N*	1445	1445	1445	1445	1445	1445

Note: ** *p* < 0.05, *** *p* < 0.01.

**Table 4:** Mediating effect of parental discipline.

	(7)	(8)	(9)	(10)	(11)	(12)
	Depression	Parental Discipline	Depression	Subjective Happiness	Parental Discipline	Subjective Happiness
One parent migrated	0.085	0.005	0.089	−0.078	0.005	−0.079
	(0.066)	(0.074)	(0.066)	(0.074)	(0.074)	(0.074)
Both parents migrated	0.150 **	−0.241 ***	0.101	−0.180 **	−0.241 ***	−0.158 **
	(0.070)	(0.077)	(0.070)	(0.077)	(0.077)	(0.077)
Parental discipline	--	--	−0.232 ***	--	--	0.096 ***
	--	--	(0.027)	--	--	(0.030)
Controls	yes	yes	yes	yes	yes	yes
School fixed effects	yes	yes	yes	yes	yes	yes
*N*	1445	1445	1445	1445	1445	1445

Note: ** *p* < 0.05, *** *p* < 0.01.

**Table 5:** Mediating effect of absolute income.

	(1)	(2)	(3)	(4)	(5)	(6)
	Depression	Absolute Income	Depression	Subjective Happiness	Absolute Income	Subjective Happiness
One parent migrated	0.085	0.093 *	0.095	−0.078	0.093 *	−0.088
	(0.066)	(0.056)	(0.067)	(0.074)	(0.056)	(0.074)
Both parents migrated	0.150 **	0.374 ***	0.191 ***	−0.180 **	0.374 ***	−0.193 **
	(0.070)	(0.059)	(0.072)	(0.077)	(0.059)	(0.079)
Absolute income	--	--	−0.098 ***	--	--	0.019
	--	--	(0.032)	--	--	(0.035)
Controls	yes	yes	yes	yes	yes	yes
School fixed effects	yes	yes	yes	yes	yes	yes
*N*	1445	1422	1422	1445	1422	1422

Note: * *p* < 0.1, ** *p* < 0.05, *** *p* < 0.01.

**Table 6:** Mediating effect of relative income.

	(7)	(8)	(9)	(10)	(11)	(12)
	Depression	Relative Income	Depression	Subjective Happiness	Relative Income	Subjective Happiness
One parent migrated	0.085	0.144 **	0.098	−0.078	0.144 **	−0.090
	(0.066)	(0.058)	(0.067)	(0.074)	(0.058)	(0.075)
Both parents migrated	0.150 **	0.401 ***	0.190 ***	−0.180 **	0.401 ***	−0.194 **
	(0.070)	(0.061)	(0.072)	(0.077)	(0.061)	(0.079)
Relative income	--	--	−0.089 ***	--	--	0.020
	--	--	(0.031)	--	--	(0.034)
Controls	yes	yes	yes	yes	yes	yes
School fixed effects	yes	yes	yes	yes	yes	yes
*N*	1445	1422	1422	1445	1422	1422

Note: ** *p* < 0.05, *** *p* < 0.01.

**Table 7:** Heterogeneity by absolute poverty.

	(1)	(2)	(3)	(4)
	Depression		Subjective Happiness	
	Poor Household	Non-Poor Household	Poor Household	Non-Poor Household
One parent migrated	0.156	0.063	0.028	−0.121
	(0.119)	(0.082)	(0.132)	(0.091)
Both parents migrated	0.268 **	0.112	−0.038	−0.233 **
	(0.130)	(0.085)	(0.143)	(0.094)
Controls	yes	yes	yes	yes
School fixed effects	yes	yes	yes	yes
*N*	446	999	446	999
chi2	66.591	58.171	64.516	72.669

Note: ** *p* < 0.05.

**Table 8:** Heterogeneity by relative poverty.

	(5)	(6)	(7)	(8)
	Depression		Subjective Happiness	
	Poor Household	Non-Poor Household	Poor Household	Non-Poor Household
One parent migrated	0.316 **	0.050	−0.229	−0.032
	(0.139)	(0.077)	(0.155)	(0.085)
Both parents migrated	0.403 ***	0.144 *	−0.205	−0.183 **
	(0.156)	(0.081)	(0.175)	(0.088)
Controls	yes	yes	yes	yes
School fixed effects	yes	yes	yes	yes
*N*	339	1106	339	1106
chi2	54.095	66.610	59.393	63.454

Note: * *p* < 0.1, ** *p* < 0.05, *** *p* < 0.01.

## Data Availability

The data presented in this study are available on request from the corresponding author.
